# Experimental and theoretical study on the propagation characteristics of stress wave in filled jointed rock mass

**DOI:** 10.1371/journal.pone.0253392

**Published:** 2021-09-09

**Authors:** Shuailong Jia, Zhiliang Wang, Jianguo Wang, Zhitang Lu, Haochen Wang

**Affiliations:** 1 School of Civil and Hydraulic Engineering, Hefei University of Technology, Hefei, Anhui, China; 2 School of Mechanics and Civil Engineering, China University of Mining and Technology, Xuzhou, Jiangsu, China; 3 School of Resource and Environmental Engineering, Hefei University of Technology, Hefei, Anhui, China; Sapienza University of Rome: Universita degli Studi di Roma La Sapienza, ITALY

## Abstract

This study is to theoretically and experimentally investigate the propagation of stress waves in the filled joint set. The time-domain recursive method is used to derive the propagation equations in the filled joint set, and the filled joints are further simplified into structural planes without joint thickness. The split-Hopkinson rock bar is modified to simulate P wave propagation normally across the parallel filled joints. The relationship among stress-closure curve, joint specific stiffness, transmission coefficient and loading rate is analyzed. The results show that, for the rock mass with a single joint, both the joint specific stiffness and transmission coefficient of different filling materials increase with loading rate. More serious particle breakage of the filling materials leads to lower joint specific stiffness and transmission coefficient. For the rock mass with two joints, the joint specific stiffness of each joint affects the transmission coefficient of the filled joint set. It is found that our theoretical calculations are basically consistent with the experimental ones, and the joint specific stiffness can well characterize the propagation behavior of stress wave in the filled parallel rock joints.

## 1. Introduction

Natural rock mass has many discontinuities such as joints, faults and bedding planes. Rock joints make the rock mass be heterogeneous [1]. They do not only affect the blast-induced stress wave propagation in the rock mass, but also have a great influence on the mechanical properties of the rock mass. The stress wave at a lower stress level cannot cause the damage of rock mass, but can produce large displacements at the joints, thus leading to the overall instability of the rock mass. On the other hand, the rock joints are generally filled by gravel, clay and other fine particle materials. These filling materials are of low strength and can deform greatly, thus playing a more significant role in the rock mass stability. Therefore, it is necessary to study the dynamic characteristics of these filling materials in fractured rock masses.

The joint property influences the mechanical behavior of fractured rock mass and the wave propagation. In recent decades, the seismic response of joints has been studied using different analytical methods, such as the displacement discontinuity method [2–4], the virtual wave source method [5–7], and the scattering matrix method [8]. Rock mass generally has multiple joints and the wave propagation thus becomes a complicated process. To simplify the problem, most of these methods focus only on non-filled joints. Such a non-filling assumption is only valid when the joints are very large in extent and small enough in thickness compared to the wavelength of an incident wave. Compared to the adjacent rocks, the filled joint is usually considered as a soft layer. Wave propagation across single joint and multiple parallel joints filled with a viscoelastic material was examined by Zhu et al [9]. They found that the transmission coefficient generally decreased with increase of joint thickness or wave frequency. The layered medium assumed that each filled joint had the similar physical and mechanical properties. This assumption was widely used in the previous analytical studies on the seismic response of multiple joints [10, 11]. However, the mechanical properties of each joint in the joint set may be dissimilar owing to the discrete and strongly heterogeneous gouges, such as joint thickness and stiffness. Therefore, the stress wave propagation in filled joint set with various properties remains further study.

The mechanical properties of rock in the zones susceptible to crack damage, such as within fault zones or volcanic edifices, can be subjected to large modification [12–14]. Stanchits et al. [15] explored the acoustic-emission (AE) triggering in an anisotropic rock and reported that the propagation of induced AE parallel to bedding plane was faster than perpendicular to bedding and related to the anisotropy of permeability. David et al. [16] experimentally investigated the effect of fluid injection on the mechanical behavior of the weakly consolidated sandstone, finding that the mechanical instability was probably linked to the loss of cohesion in the water-invaded region. The physical characterization of carbonate-bearing normal faults was fundamental for the resource development and seismic hazard. Trippetta et al. [17, 18] explored the petrophysical properties of carbonate rock and concluded that the physical and transport properties of the investigated fault zone were controlled by different deformation processes. Rajabzadeh et al. [19] determined the effect of rock class and porosity on the relationship between uniaxial compressive strength (UCS) and some other properties of the carbonate rock with different geneses, showing that utilizing porosity did not yield any significant improvement of correlation coefficients between main variables and UCS. In addition, many studies have been done on the effect of joint on wave propagation. Sound velocity tests were carried out on rock samples with different fracture roughness by Kahraman [20], who found that the sound velocity varied with fracture roughness. Kurtulus et al [21] investigated the effect of parallel and variable directional joint on ultrasonic pulse propagation, indicating that the attenuation velocity was higher in the prismatic marble with variable directional joints. Leucci and De Giorgi [22] experimentally explored the effect of joint on the propagation velocities of P-wave and S-wave passing through parallel joints, and concluded that the joint acted as filter by attenuating the high frequency components in the spectrum of the waveform. Among various experimental methods, the Split-Hopkinson Pressure Bar (SHPB) has been widely used to study the dynamic properties of joint [23–26]. The conventional SHPB was improved to investigate the dynamic property of joint by using rock bars as input and output bars [27–29]. As stated above, previous studies on the dynamic characteristics of rock mass joints mostly focused on the same joint-filling material and loading rate, and the physical and mechanical properties of each joint were considered identically. However, the different loading rates and joint-filling materials lead to dissimilar physical and mechanical properties of the filled joint in a joint set, and the question remains open to the dynamic characteristics of rock mass joints.

This study is to investigate the propagation of stress wave in the filled joint set. At first, the time domain recursion method is extended to derive the stress wave propagation equation in the filled joint set, and the filled joint is further simplified into the structural plane without thickness. Subsequently, the modified Split–Hopkinson Rock Bar (SHRB) is used to simulate the P-wave propagation in the filled joint set, and the dynamic mechanical properties of filling materials and the propagation of stress wave are analyzed. Finally, the transmitted wave is calculated in the time domain to compare with the experimental results.

## 2. Analytical method

### 2.1 Filled joint model

**Fig 1:**
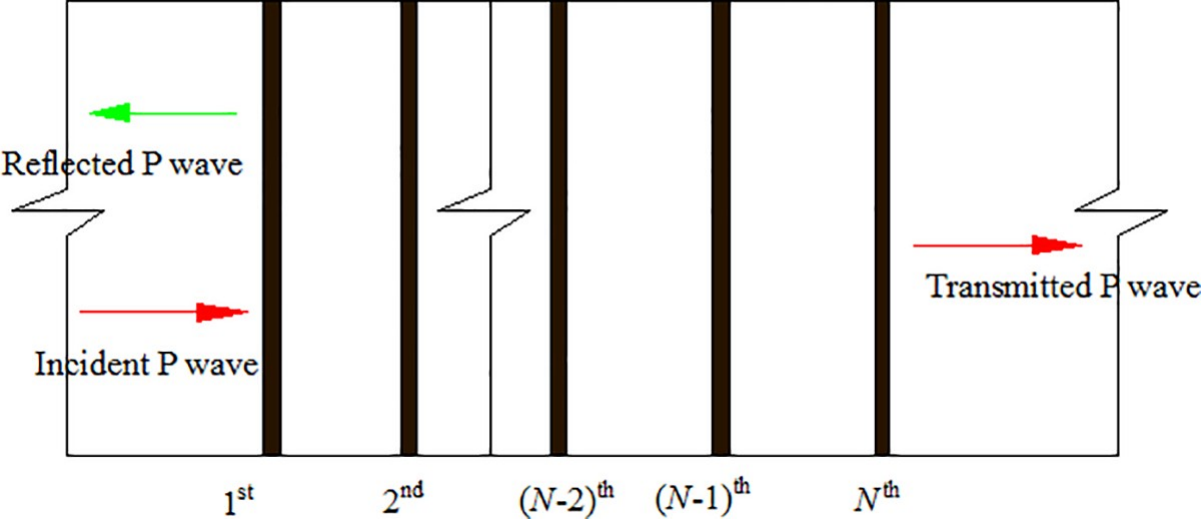
Incident P wave upon a rock mass with a set of parallel filled joints.

We consider a homogeneous, isotropic and linear rock. The rock contains a set of parallel filled joints, as shown in Fig 1, where *N* denotes the joint number. Each filled joint is planar, large in extent and small in thickness compared to the wavelength. The joint material is equivalent as an elastic and homogeneous medium which is different from the rock.

**Fig 2:**
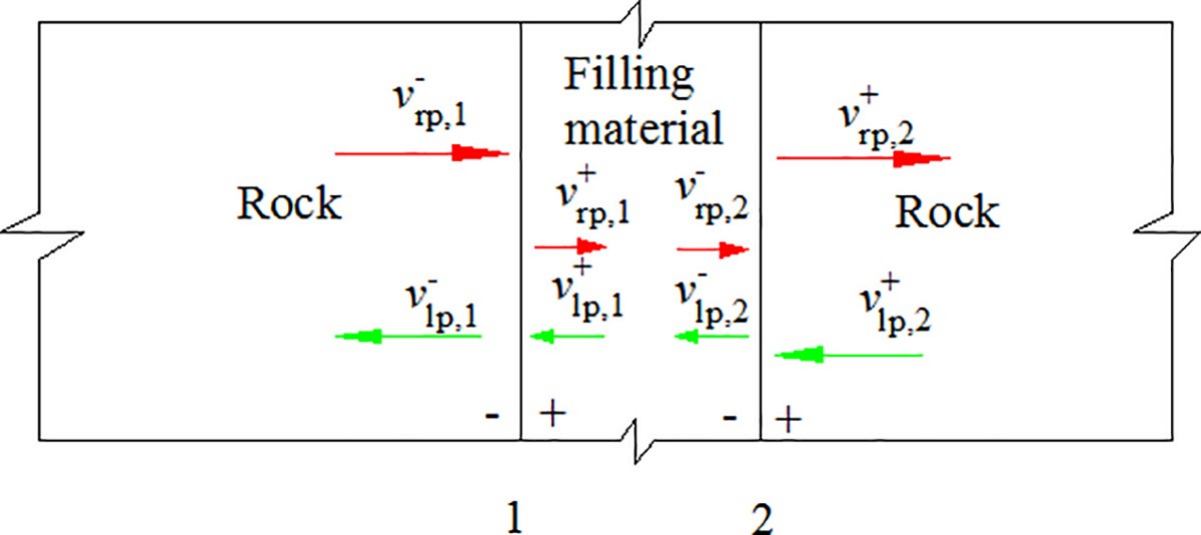
Filled joint model.

The filled joint is considered as a thin-layer interface between two intact rocks. As shown in Fig 2, the thin-layer is composed of displacement continuous surface 1, filling material and displacement continuous surface 2. When the stress wave propagates to the two sides of the filled joint, both reflection and transmission take place. *v*_rp_ and *v*_lp_ are the particle velocity of the right-running and left-running P wave, respectively.

### 2.2 Propagation equation of stress wave

The present investigation is to study the stress wave propagation in a rock mass with a set of parallel filled joints by using time-domain recursive method. According to the literature [30], the expressions of the normal stress before and after the contact interface 1 are obtained as

$$\{\begin{array}{c} \sigma^{-}=z_{pr}v_{rp,1}^{-}+z_{pr}v_{lp,1}^{-}\\ \sigma^{+}=z_{pf}v_{rp,1}^{+}+z_{pf}v_{lp,1}^{+} \end{array}$$ (1)

where the superscript “-” and “+” represent the left and right sides of the joint contact interface, respectively. *z* is the P wave impedance and *z* = *ρc*, *c* is the P wave propagation velocity in the medium, *ρ* is the density. *z*_pr_ and *z*_pf_ are the P wave impedance of the rock and the filling material, respectively. The normal components, $v_{n}^{-}$ and $v_{n}^{+}$, of the velocity before and after the contact interface are expressed as

$$\{\begin{array}{c} v_{n}^{-}=v_{rp,1}^{-}-v_{lp,1}^{-}\\ v_{n}^{+}=v_{rp,1}^{+}-v_{lp,1}^{+} \end{array}$$ (2)

For the filled joint J, the joint contact interface satisfies the continuity of stress and displacement as

$$\{\begin{array}{c} \sigma^{-}=\sigma^{+}\\ v_{n}^{-}=v_{n}^{+} \end{array}$$ (3)

Substituting Eqs (1) and (2) into Eq (3) yields:

$$A{\left[\begin{array}{c} v_{rp,1}^{-}\\ v_{lp,1}^{-} \end{array}\right]}_{J}=B{\left[\begin{array}{c} v_{rp,1}^{+}\\ v_{lp,1}^{+} \end{array}\right]}_{J}$$ (4)

where *A* and *B* are the matrix parameters and written as

$$A=[\begin{array}{cc} z_{pr} & z_{pr}\\ 1 & -1 \end{array}]\;B=[\begin{array}{cc} z_{pf} & z_{pf}\\ 1 & -1 \end{array}]$$

When the stress wave passes through contact interface 1, the particle velocity can be obtained from Eq (4). The filling material is an elastic and homogeneous medium, thus the time-shifting function should be satisfied. Therefore, when the stress wave propagates to the filled joint interface 2, the expression is

$$\{\begin{array}{l} v_{rp,2}^{-}(t)=v_{rp,1}^{+}(t-\Delta t_{pf})\\ v_{lp,1}^{+}(t)=v_{lp,2}^{-}(t-\Delta t_{pf}) \end{array}$$ (5)

$$B{\left[\begin{array}{c} v_{rp,2}^{-}\\ v_{lp,2}^{-} \end{array}\right]}_{J}=A{\left[\begin{array}{c} v_{rp,2}^{+}\\ v_{lp,2}^{+} \end{array}\right]}_{J}$$ (6)

where Δ*t*_pf_ is the propagation time of P-wave in the filled joint.

If the distance between adjacent filled joints is *l*, the expression of the stress wave between adjacent filled joints can be expressed as

$$\{\begin{array}{l} v_{rp,1}^{-}{\left(t\right)}_{J}=v_{rp,2}^{+}{\left(t-\Delta t_{pr}\right)}_{J-1}\\ v_{lp,2}^{+}{\left(t\right)}_{J-1}=v_{lp,1}^{-}{\left(t-\Delta t_{pr}\right)}_{J} \end{array}$$ (7)

where Δ*t*_pr_ is the propagation time of the stress wave between adjacent joints. The propagation law of stress wave in the filled joint set can be analyzed by Eqs (4)–(7).

**Fig 3:**
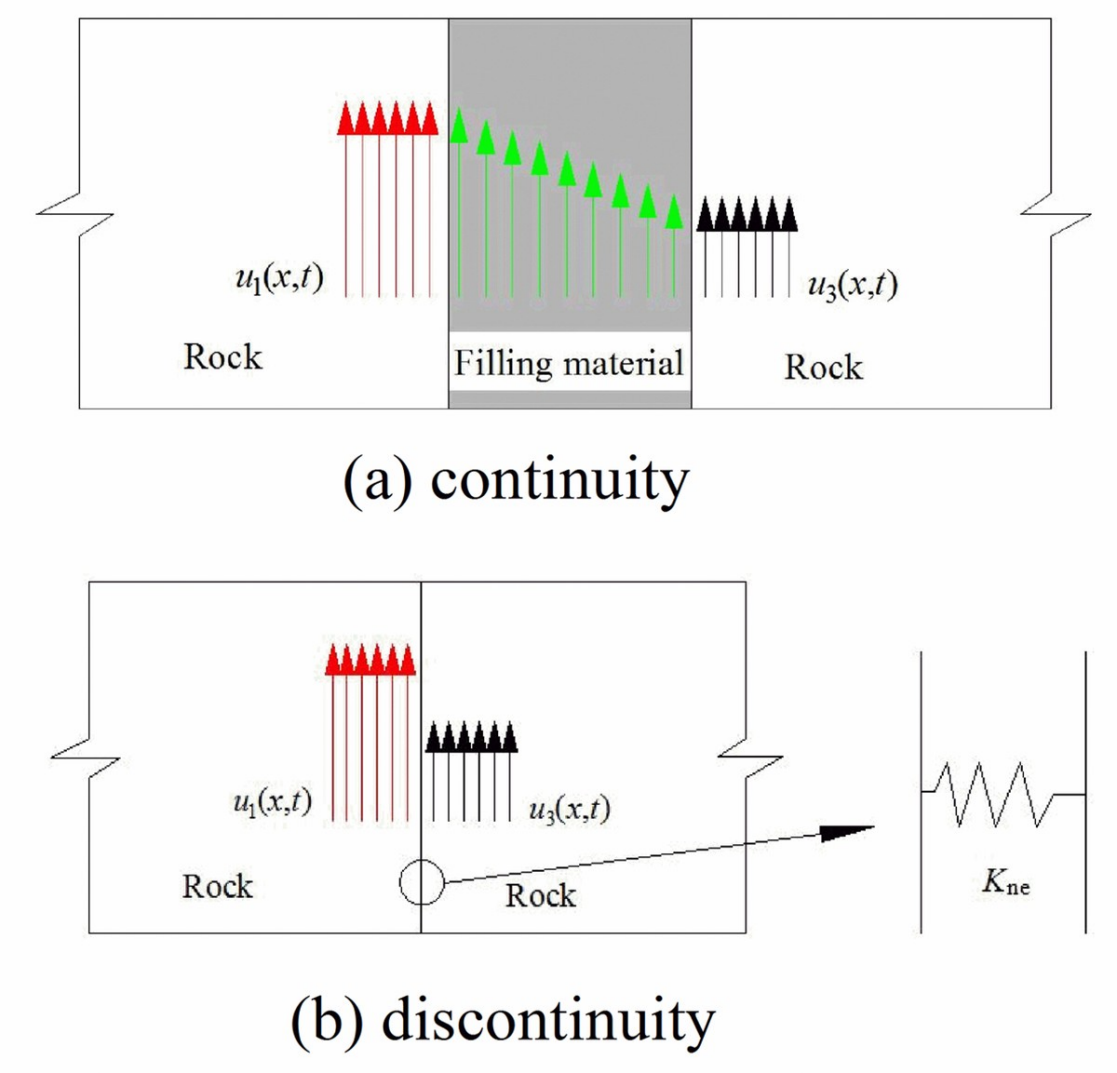
Schematic view of the displacement at the filled joint. (a) Continuity (b) Discontinuity.

When the stress wave propagates in the rock mass, the physical properties of the filling material are difficultly determined. Meanwhile, the width of the joint is smaller than the wave length of the stress wave. Therefore, the filled joint can be equivalent to a discontinuous displacement model. When stress wave propagates across a filled joint with one thin thickness, the displacement at each side of the filled joint is continuous, as shown in Fig 3(A). If the filled joint is assumed as a discontinuous displacement model with zero-thickness, there is a distinct jump in the displacement at the zero-thickness interface, which is modeled as the displacement discontinuity boundary condition and shown in Fig 3(B).

### 2.3 Equivalent normal stiffness

Based on the assumption of stress uniformity, during the propagation of the stress wave in the filled joint, the stress and joint closure can be deduced through the particle velocity before and after the filled joint. The expressions of stress and joint closure are

$$\sigma {\left(t\right)}_{J}=\frac{E_{r}}{2c_{r}}{\left[v_{rp,1}^{-}(t)+v_{lp,1}^{-}(t)+v_{rp,2}^{+}(t)+v_{lp,2}^{+}(t)\right]}_{J}$$ (8)

$$\Delta l{\left(t\right)}_{J}=\int [v_{rp,1}^{-}(t)-v_{lp}^{-}(t)-(v_{rp,2}^{+}(t)+v_{lp,2}^{+}(t)){]}_{J}$$ (9)

where *E*_r_ is the elastic modulus of the rock. *c*_r_ is the propagation speed of P wave in the rock.

**Fig 4:**
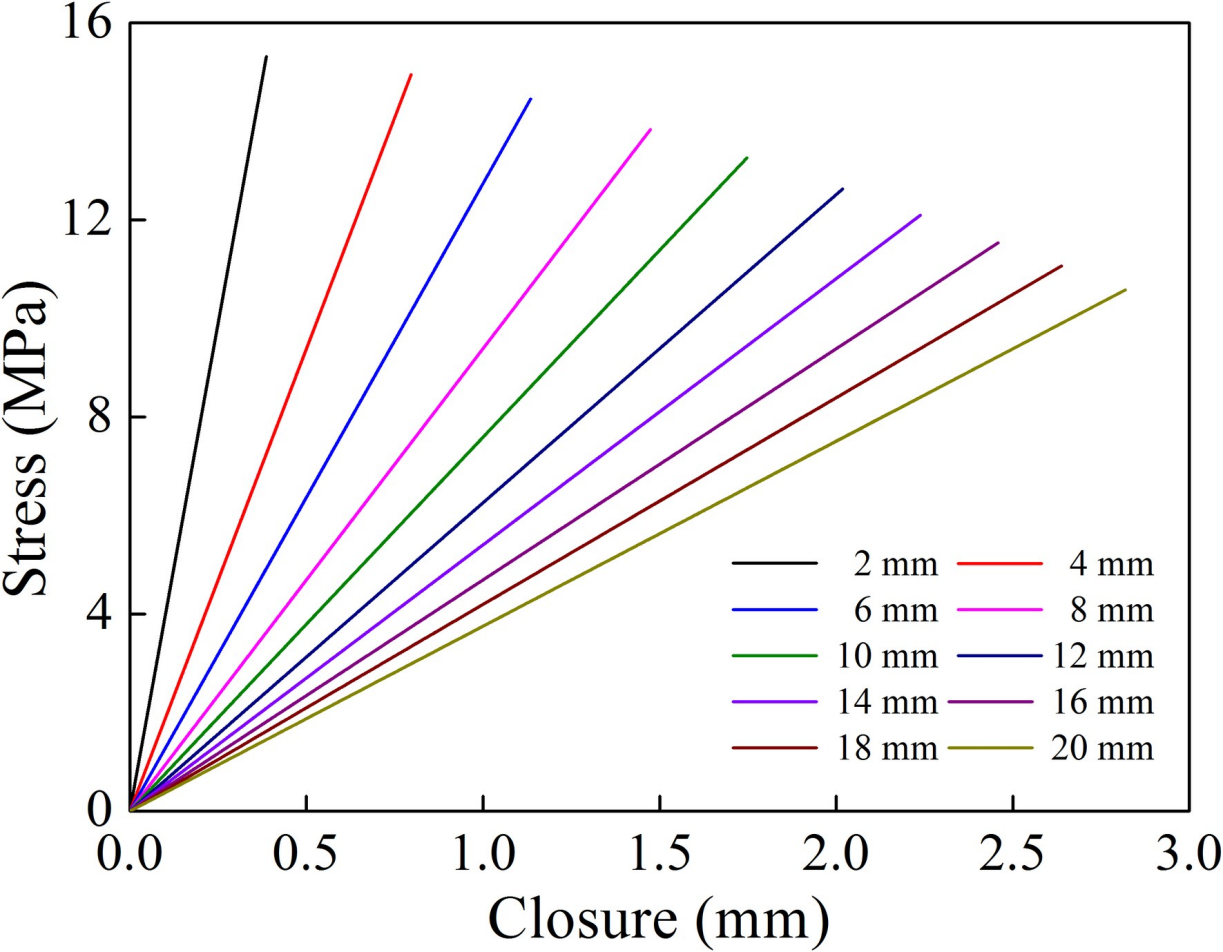
Relationship between normal stress and closure (a single filled joint).

**Fig 5:**
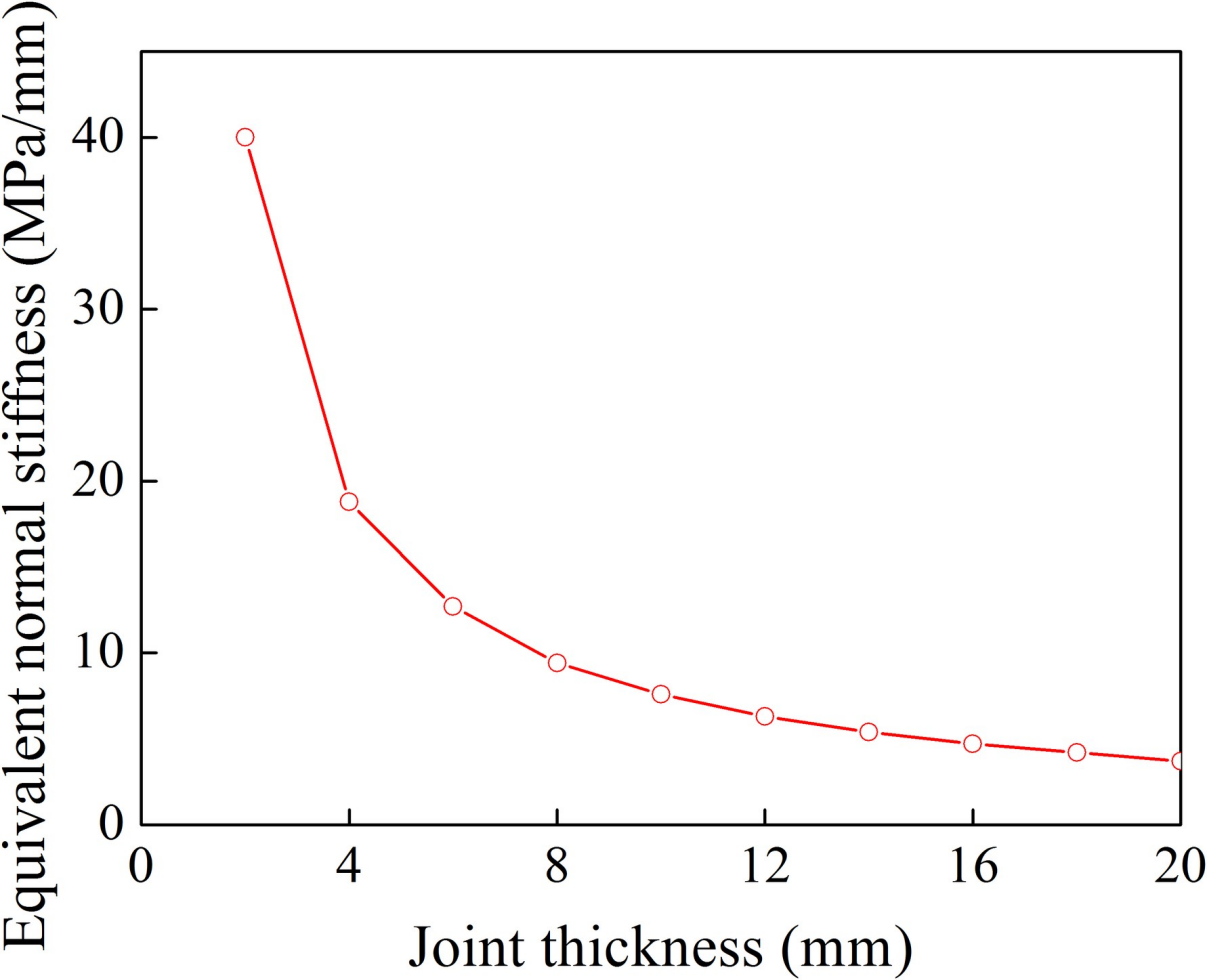
Equivalent normal stiffness of filled joint.

When a P-wave normally impinges a filled joint, the reflected and transmitted waves can be calculated by Eqs (4)–(6). The normal stress and the closure of the filled joint can be obtained from Eqs (8) and (9). In the following analysis, it is assumed that the rock density *ρ*_r_ is 2650 kg/m^3^, the P wave velocity in the rock is 5830 m/s. The filling material density *ρ*_f_ and P wave velocity are 1700 kg/m^3^ and 210 m/s, respectively. The incident wave is assumed to be in harmonic waveform with the amplitude 1 m/s [31]. The relationship between the normal stress and the closure is shown in Fig 4 for eight joint thicknesses. It is observed that for a given joint thickness the closure of the filled joint linearly increases with the increase of normal stress. The equivalent normal stiffness is defined as the ratio of the normal stress to the joint closure. Fig 4 shows that the variation of the equivalent normal stiffness with the filled joint thickness can be obtained. As shown in Fig 5, the equivalent normal stiffness drops sharply first for smaller thickness and then decrease smoothly with the increase of filled joint thickness.

The equivalent normal stiffness *K*_ne,J_ can be obtained after Eq (8) is combined with Eq (9), and Eqs (4)–(7) can also expressed as

$$v_{rp}^{-}{\left(i\right)}_{J}+v_{lp}^{-}{\left(i\right)}_{J}=v_{rp}^{+}{\left(i\right)}_{J}+v_{lp}^{+}{\left(i\right)}_{J}$$ (10)

$$C{\left[\begin{array}{c} v_{rp}^{+}(i+1)\\ v_{lp}^{+}(i+1) \end{array}\right]}_{J}=D{\left[\begin{array}{c} v_{rp}^{-}(i)\\ v_{lp}^{-}(i) \end{array}\right]}_{J}+E{\left[\begin{array}{c} v_{rp}^{+}(i)\\ v_{lp}^{+}(i) \end{array}\right]}_{J}$$ (11)

where *C*, *D* and *E* are the matrix parameters. They are written as

$$C=[\begin{array}{c} K_{ne,J}\Delta t\\ K_{ne,J}\Delta t \end{array}]\;D=[\begin{array}{c} z_{pr}-K_{ne,J}\Delta t\\ z_{pr}-K_{ne,J}\Delta t \end{array}]\;E=[\begin{array}{c} z_{pr}\\ z_{pr} \end{array}]$$

where *K*_ne,J_ is the equivalent normal stiffness of the *J* filled joint.

## 3. Test preparation

### 3.1 Test device

**Fig 6:**
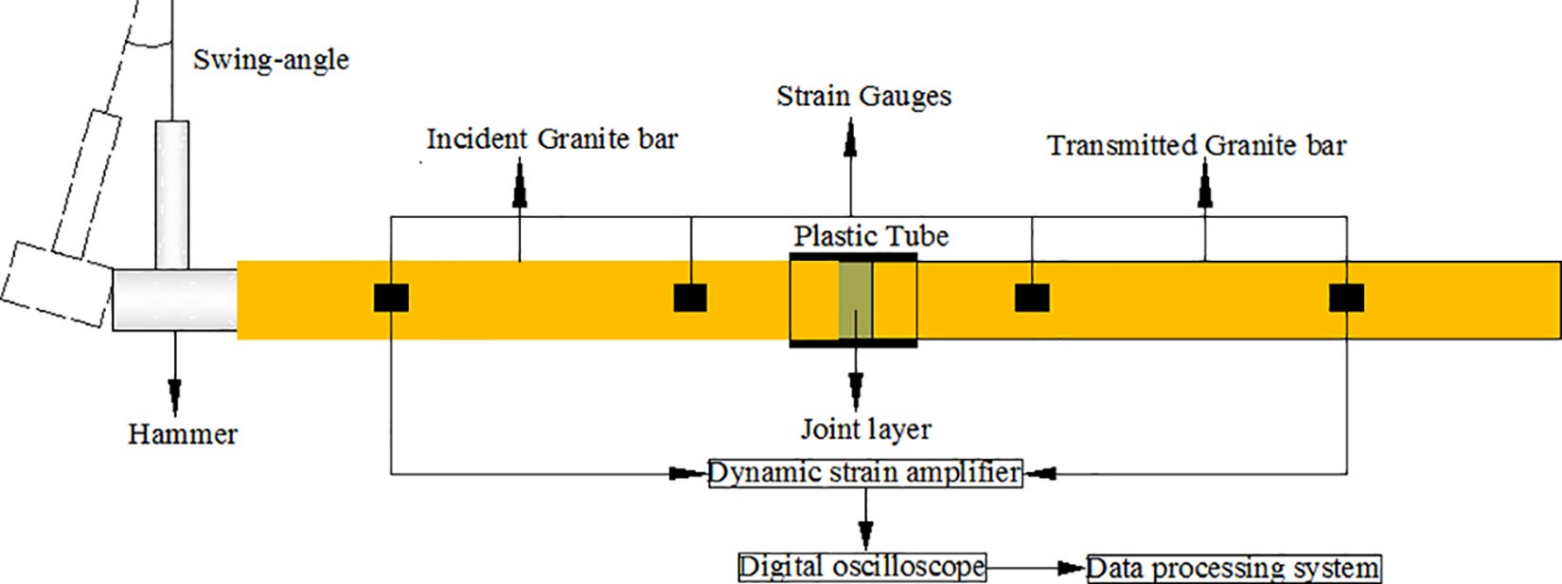
SHRB device.

The SHRB is shown in Fig 6. It was mainly composed of pendulum, incident and transmitted rock bars. The pendulum was made of high-strength steel with a diameter of 70mm. The incident and transmitted rock bars were made of granite that was taken from the Huashan area (Shanxi Province, northwest China). The bars were 985 mm in length and 70 mm in diameter. The wave speed *c* of the rock bar was 3300 m/s, the density *ρ* was 2650 kg/m^3^, and the elastic modulus *E* was 28.86 GPa. In order to ensure the integrity and homogeneity of the rock bar, the ultrasonic instrument was used to carefully screen the rock bar. This can reduce the effect of the internal defects of the rock bar on test results. Further, the flatness and parallelism of the two ends of the rock bar were elaborately controlled to avoid stress concentration due to the bias of the rock bar during loading stage.

The data acquisition unit was configured based on the LabVIEW platform, including signal triggering, data recording and storage. Two groups of strain gauges were attached to the incident and transmitted rock bars and were connected in a Wheat-stone full-bridge to average out bending strain. The distance between the strain gauge and the bar end should be greater than five times the diameter of the bar so as to eliminate the influence of radial inertia effect [32]. The stress wave measured in the rock bar was superposed by incident wave and reflected wave. To facilitate the separation of stress wave, the position of the strain gauge should be as close as possible to the end surface of the rock bar. Therefore, the selected strain gauge positions were 35 cm and 60 cm away from the bar end O, respectively.

### 3.2 Joint-filling material

**Fig 7:**
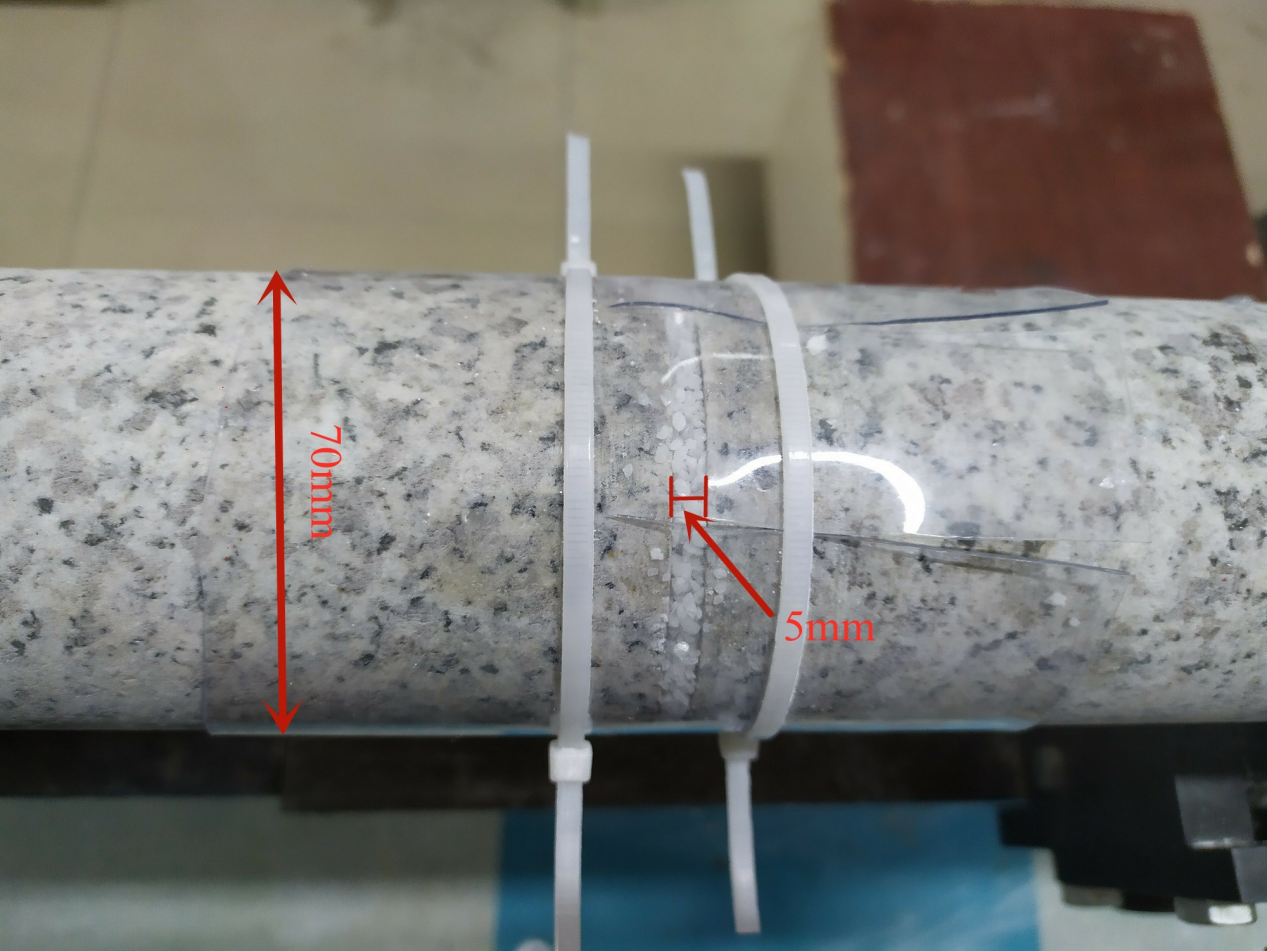
Filling material between the incident and transmitted rock bar.

**Fig 8:**
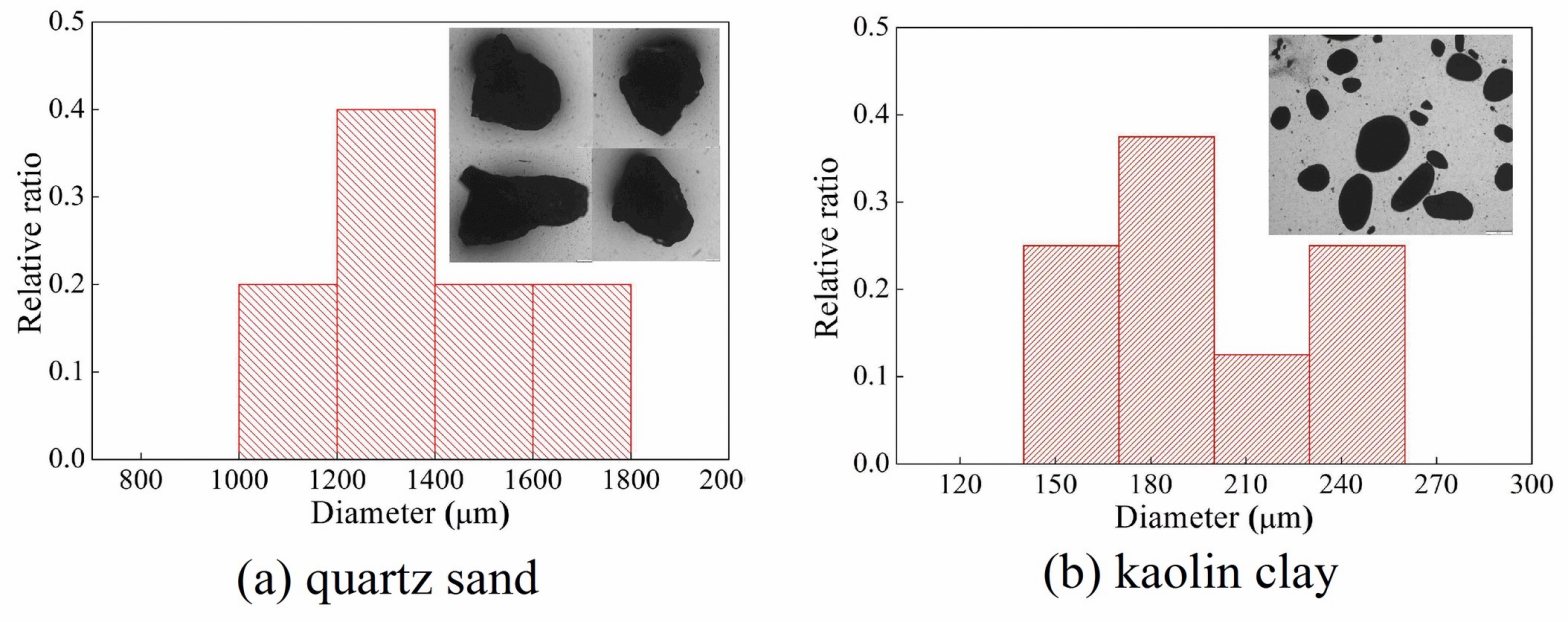
Joint-filling materials. (a) quartz sand (b) kaolin clay.

The joint-filling material was sandwiched between the incident and transmitted rock bar during the test. It was packed in a plastic tube to prevent outflow. At this time, the joint-filling material was in a uniaxial strain state (see Fig 7). In the rock mass test of single and two joints, the joint-filling materials were made up of quartz sand, kaolin clay and their mixtures, see Fig 8. The diameter of quartz sand particles was 1-2mm, and the particle size of kaolin clay was about 0.2 mm. There were two main reasons for choosing quartz sand as the filling material: (1) The moisture content of quartz sand at room temperature was negligible, and its viscosity was basically zero. (2) Quartz sand was composed of a pure mineral, and the physical properties of quartz sand were relatively stable in each test. Kaolin clay was the main component of clay in natural faults and its lattice parameters did not change when it was wetted [33]. For the mixture of quartz sand and kaolin clay, the kaolin clay accounts for 50% in the weight of the mixture. The quartz sand and kaolin clay were firstly tumbled with pure water and were shaken manually, until the mixture was visually homogeneous, and the mixture was maintained at a room condition for approximately 24h.

### 3.3 Date processing method

**Fig 9:**
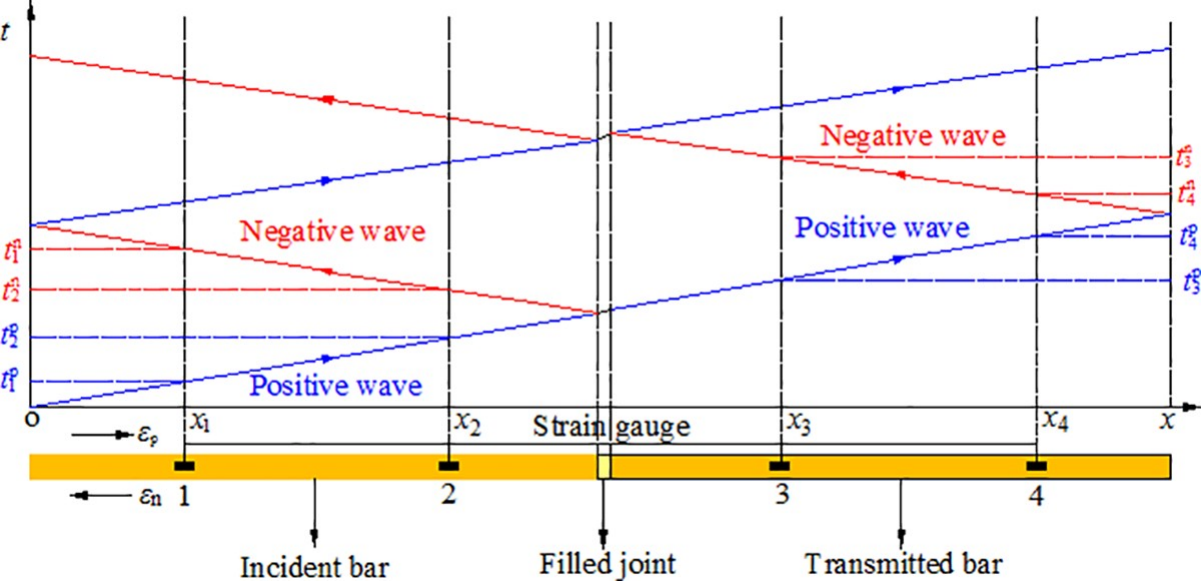
Schematic diagram of the wave separation in rock bar.

One-dimensional stress wave theory is applicable to the rock bar if the wave length of stress wave is larger than the rock bar diameter during the stress wave propagation in the rock bar. For convenience, the compressive stress is positive and the tensile stress is negative. The test recording of these two strain gauges are *ε*_1_(*t*) and *ε*_2_(*t*) which are superposed by two stress waves in opposite directions, $\varepsilon_{1}^{p}(t)$ and $\varepsilon_{1}^{n}(t)$ for gauge 1, $\varepsilon_{2}^{p}(t)$ and $\varepsilon_{2}^{n}(t)$ for gauge 2, respectively, where the symbols p and n represent the positive and negative directions, see Fig 9.

We define

$$t_{1}=x_{1}/c,\;t_{2}=x_{1}/c+(x_{2}‐x_{1})/c,\;t_{3}=x_{1}/c+(x_{2}‐x_{1})/c+2\cdot (l‐x_{2})/c,\;t_{4}=x_{1}/c+2\cdot (x_{2}‐x_{1})/c+2\cdot (l‐x_{2})/c.$$

where *x*_1_ and *x*_2_ are the distances of the strain gauges 1 and 2 from the rock bar end O. *l* is the rock bar length. $\varepsilon_{1}^{p}(t)$, $\varepsilon_{1}^{n}(t)$, $\varepsilon_{2}^{p}(t)$ and $\varepsilon_{2}^{n}(t)$ can be obtained by the following time-shift function [32]:

$$\varepsilon_{1}^{p}(t)=[\varepsilon_{1}(t)-\varepsilon_{2}^{n}(t-t_{0})]\times H_{1}(t-t_{1})$$ (12)

$$\varepsilon_{2}^{p}(t)=\varepsilon_{1}^{p}(t-t_{0})\times H_{2}(t-t_{2})$$ (13)

$$\varepsilon_{2}^{n}(t)=[\varepsilon_{2}(t)-\varepsilon_{2}^{p}(t)]\times H_{3}(t-t_{3})$$ (14)

$$\varepsilon_{1}^{n}(t)=\varepsilon_{2}^{n}(t-t_{0})\times H_{4}(t-t_{4})$$ (15)

when *t* < *t*_4_, $\varepsilon_{2}^{n}(t-t_{0})=0$. *H*_i_(*t*) is the Heaviside function as

$$H_{i}(t)=\{\begin{array}{c} 1,t\geq t_{i}\\ 0,t<t_{i} \end{array}\;(i=1,2,3,4)$$ (16)

The positive wave and the negative wave at any section *x* in the rock bar can be obtained by time shifting the positive wave of gauge 1 and the negative wave of gauge 2, respectively.


$$\{\begin{array}{c} \varepsilon_{x}^{p}(t)=\varepsilon_{1}^{p}(t-\frac{x-x_{1}}{c})\\ \varepsilon_{x}^{n}(t)=\varepsilon_{2}^{n}(t-\frac{x_{2}-x}{c}) \end{array}$$ (17)

The strain at any section *x* is

$$\varepsilon_{x}(t)=\varepsilon_{x}^{p}(t)+\varepsilon_{x}^{n}(t)$$ (18)

According to the one-dimensional stress wave theory, the expressions of the stress at the joint and the joint closure are [34]

$$\sigma (t)=E(\varepsilon^{p-}(t)+\varepsilon^{p+}(t))$$ (19)

$$\Delta u(t)=c\int_{0}^{t}\left[\left(\varepsilon^{p-}(t)-\varepsilon^{n-}(t))-(\varepsilon^{p+}(t)-\varepsilon^{n+}(t)\right]\right)dt$$ (20)

where *ε*^p−^(*t*) and *ε*^n−^(*t*) are the positive and negative waves on the left side of the filled joint, respectively. *ε*^p+^(*t*) and *ε*^n+^(*t*) are the positive and negative waves on the right side of the filled joint, respectively.

**Fig 10:**
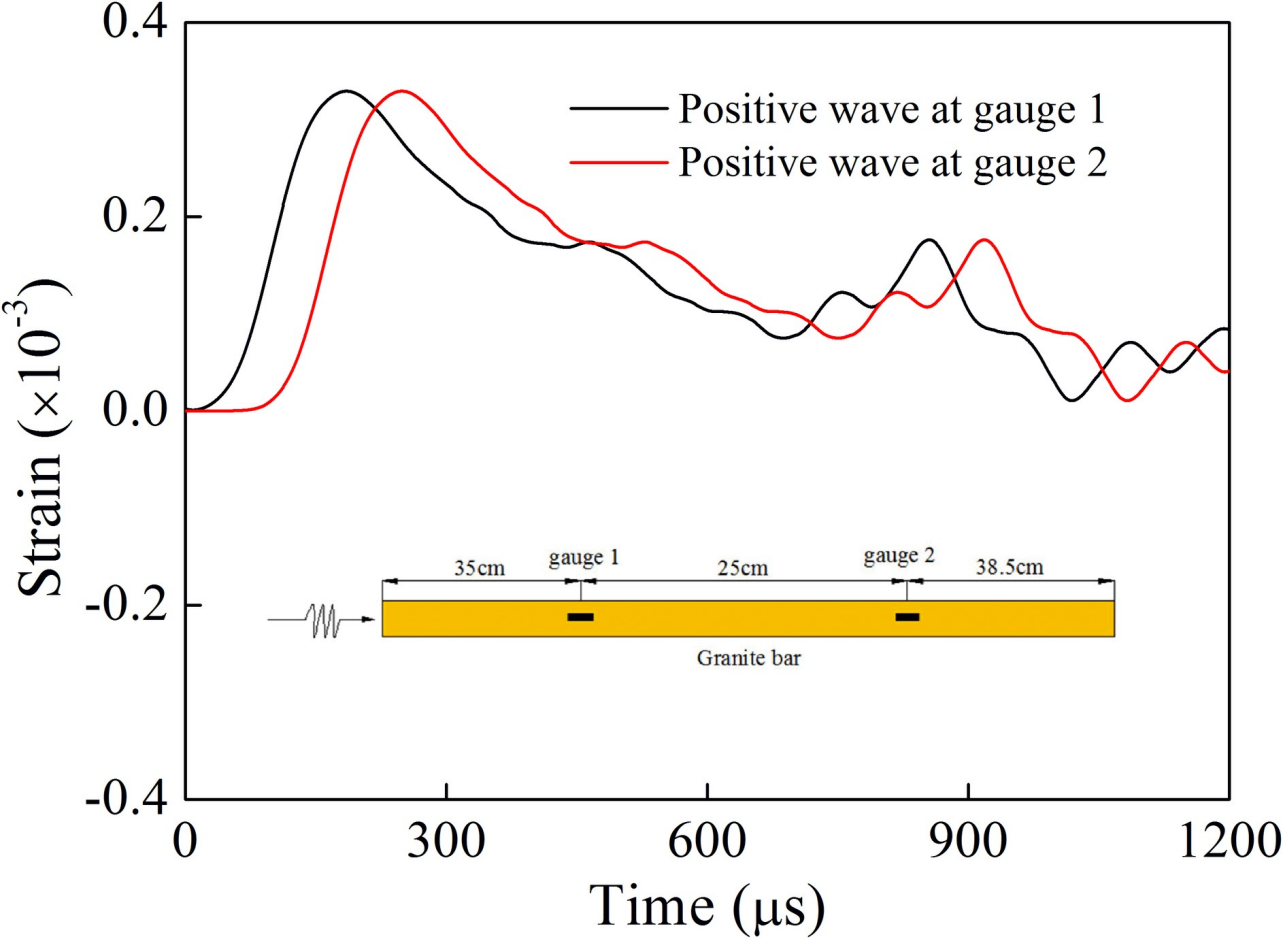
The strain time history at gauge 1 and 2 (52.0 GPa/s).

Since the rock bar is relatively long (985 mm), it is necessary to analyze the attenuation degree of stress wave in the rock bar. Taking the rock bar with a single joint as an example, the filling material was quartz sand, and the loading rate was 52.0 GPa/s. According to the stress wave separation method [32], the positive wave of the incident rock bar at gauge 1 and 2 could be obtained, see Fig 10. If the dynamic load was applied at the end of the incident bar, the peak values of the positive wave at gauge 1 and 2 were basically similar, and the positive wave had no evident attenuation. Therefore, the attenuation of stress wave in the rock bar propagation could be ignored, and the attenuation of stress wave was mainly caused by the filled joint.

## 4. Experimental results and analysis

### 4.1 Effect of loading rate on stress time-history curve

**Fig 11:**
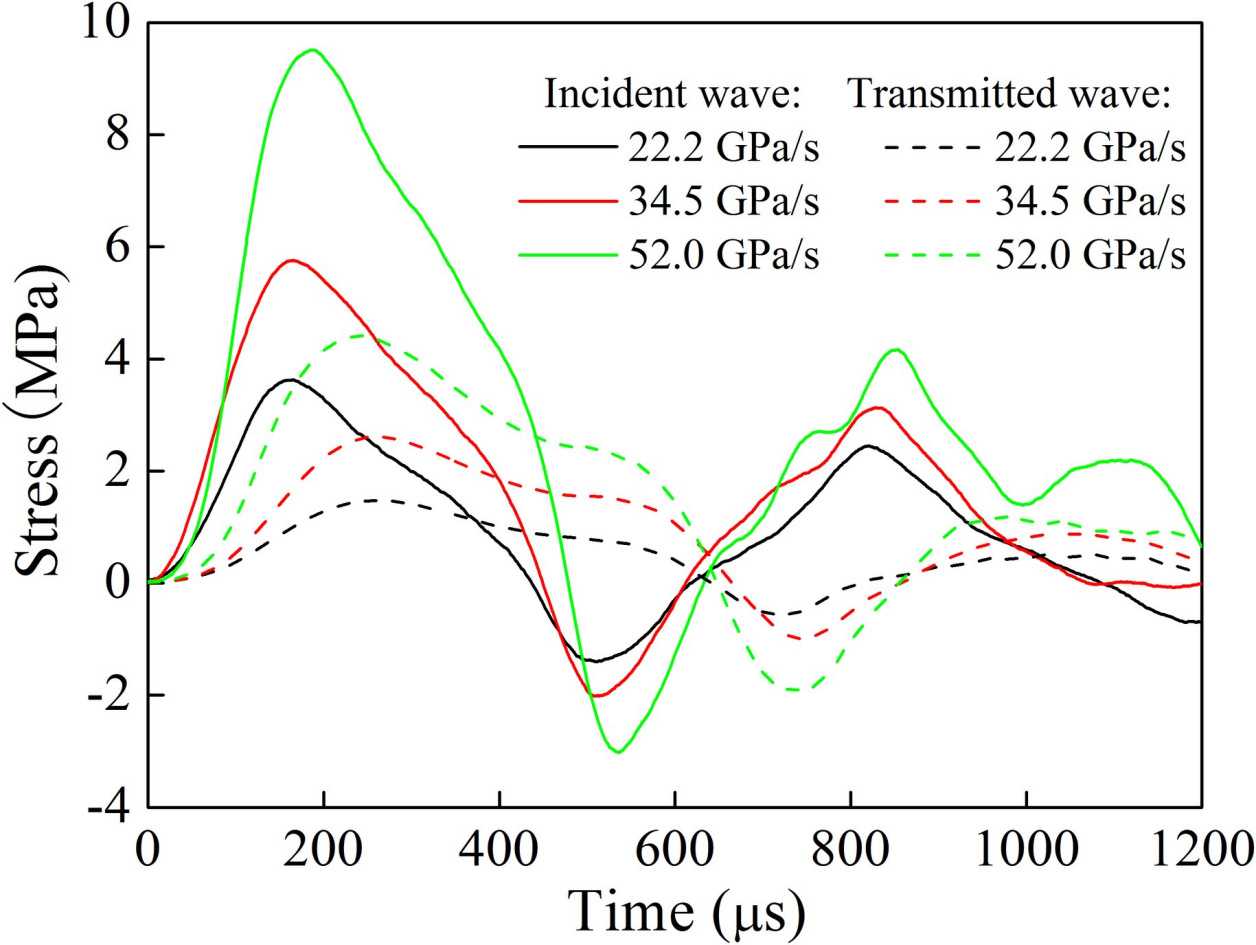
Stress time history curve in the bar with a single joint.

**Fig 12:**
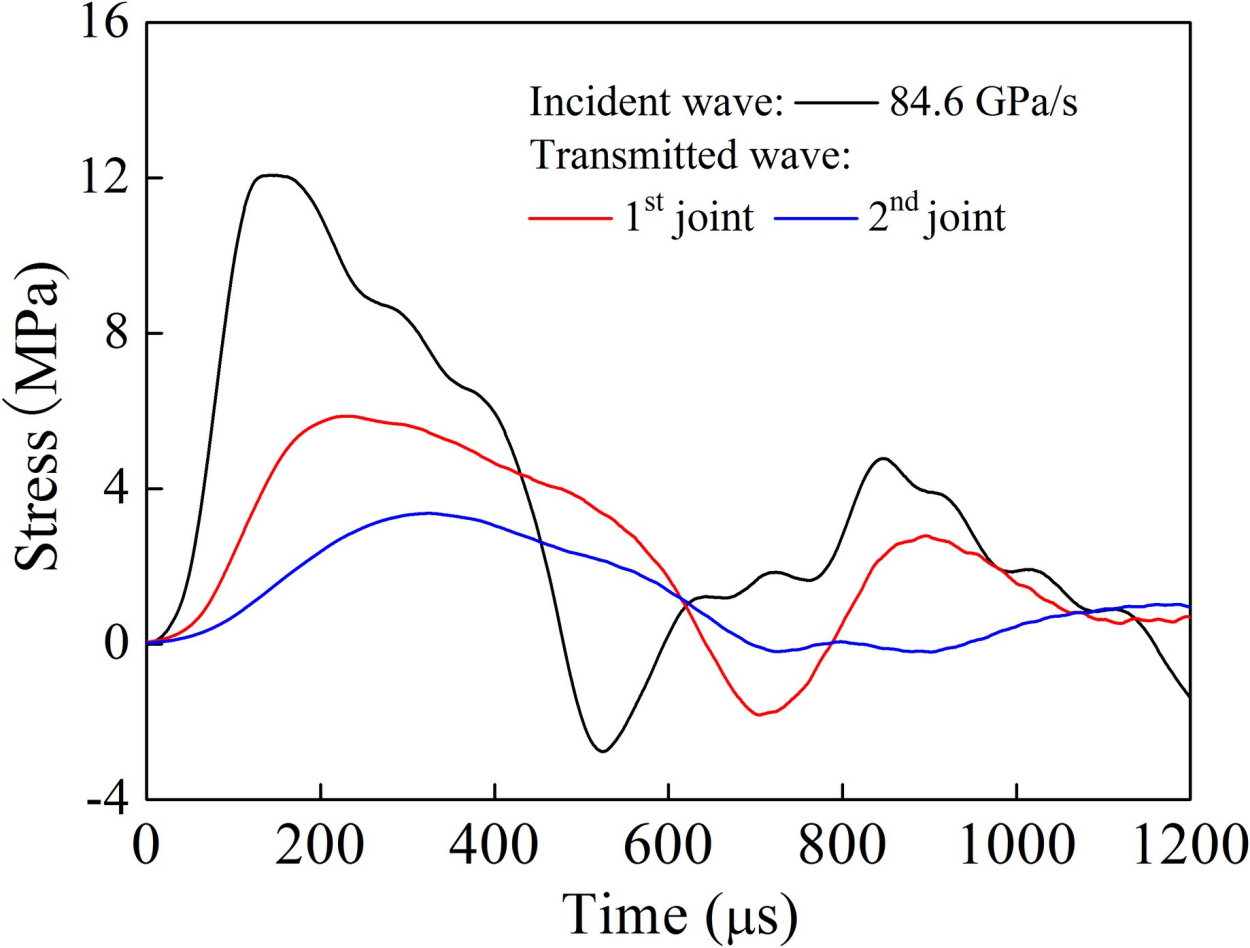
Stress time history curves in the bar with two joints (*d*_1_ = *d*_2_ = 3 mm).

The loading rate is defined as the ratio of peak stress to its corresponding time. In the rock mass test with a single joint, the pendulum angle was set to 10°, 15° and 20°, respectively. The corresponding loading rate in the incident bar end was 22.2 GPa/s, 34.5 GPa/s and 52.0 GPa/s, respectively. Meanwhile, the filled joint thickness was 3 mm, 4 mm and 5 mm, respectively. For the rock mass with two joints, the pendulum angle was set to 25°, 30° and 35°, respectively. The corresponding loading rate in the incident bar end was 74.7 GPa/ s, 77.8 GPa/s and 84.6 GPa/s, respectively. Fig 11 shows the stress time-history curve in the bar with a single joint (*d* = 3 mm) when quartz sand filling material is taken as an example. It can be found that when the joint thickness was the same, the shape of the incident stress wave under different loading rates was basically similar, and the peak value of transmitted stress wave increased with the increase of loading rate. For the rock mass test with two joints (*d*_1_ = *d*_2_ = 3 mm), the stress time-history curve is shown in Fig 12. It is observed that the peak stress was significantly reduced, when the stress wave passed through the first and second joints.

### 4.2 Test results for a single joint

#### 4.2.1 Stress-closure relationship

**Fig 13:**
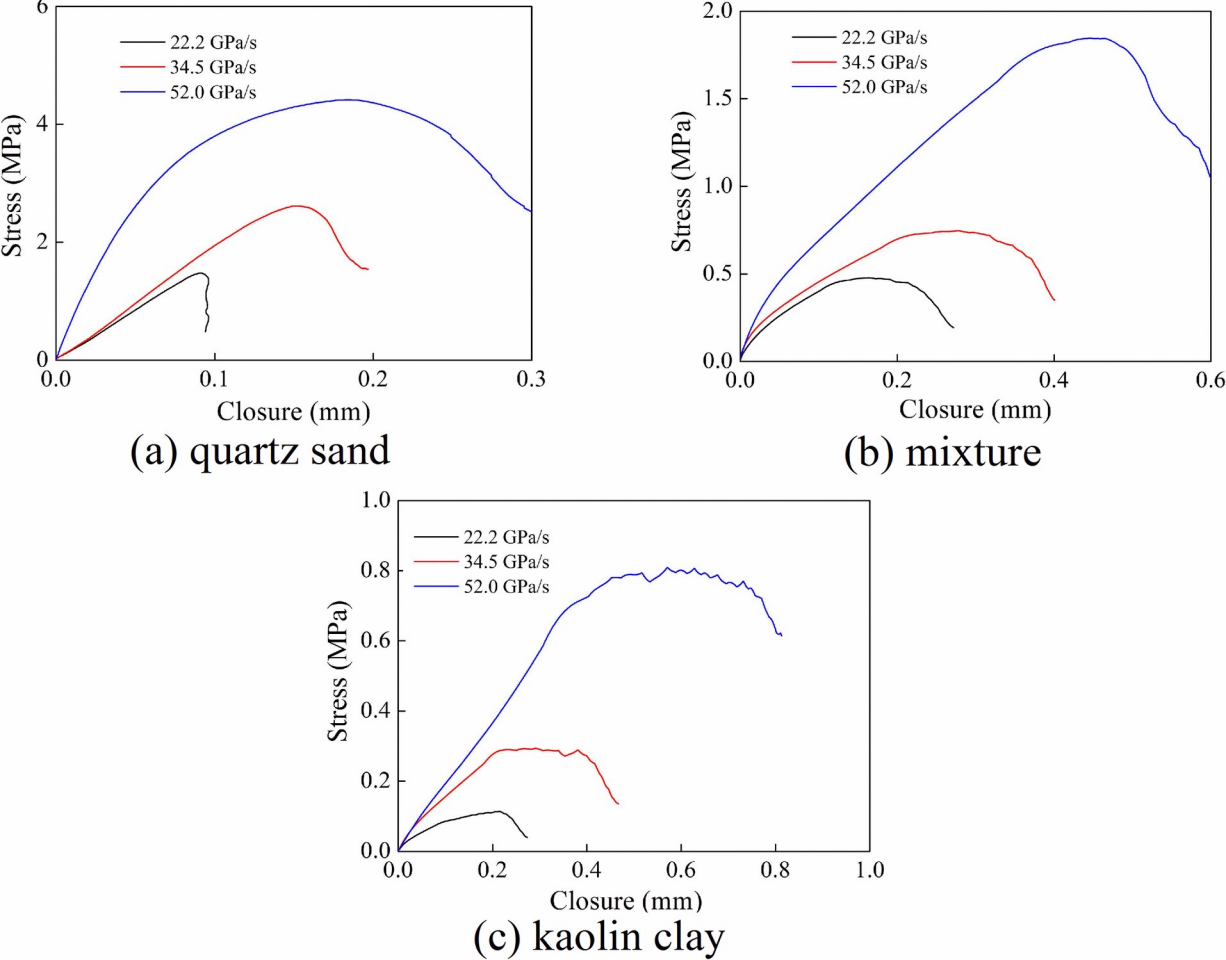
Stress-closure relationship at different loading rates (*d* = 3 mm). (a) quartz sand (b) mixture (c) kaolin clay.

Fig 13 plots the stress versus normalized closure responses of the joints. The stress-closure curves of different filling materials exhibited linearity at the initial stage of loading, and this linearity at the loading initial stage became gradually evident with the increase of loading rate. Then, the tangential slope of the stress-closure curve decreased with augmented stress. At this time, the particles of the filling material gradually slide or break until the stress in the filling material reached the peak value. Finally, as the stress decreased, the joint closure gradually increased. At the same loading rate, the quartz sand particles were basically not broken. Therefore, the linearity of the stress-closure curve in the loading stage was more evident. For the mixture material (quartz sand and kaolin clay) and kaolin clay, the kaolin clay particles were easily broken under impact load [27]. Thus, the load transfer capacity of the kaolin clay particles was reduced, and the stress-closure curve at the loading stage showed less evident linearity.

#### 4.2.2 Joint special stiffness

**Fig 14:**
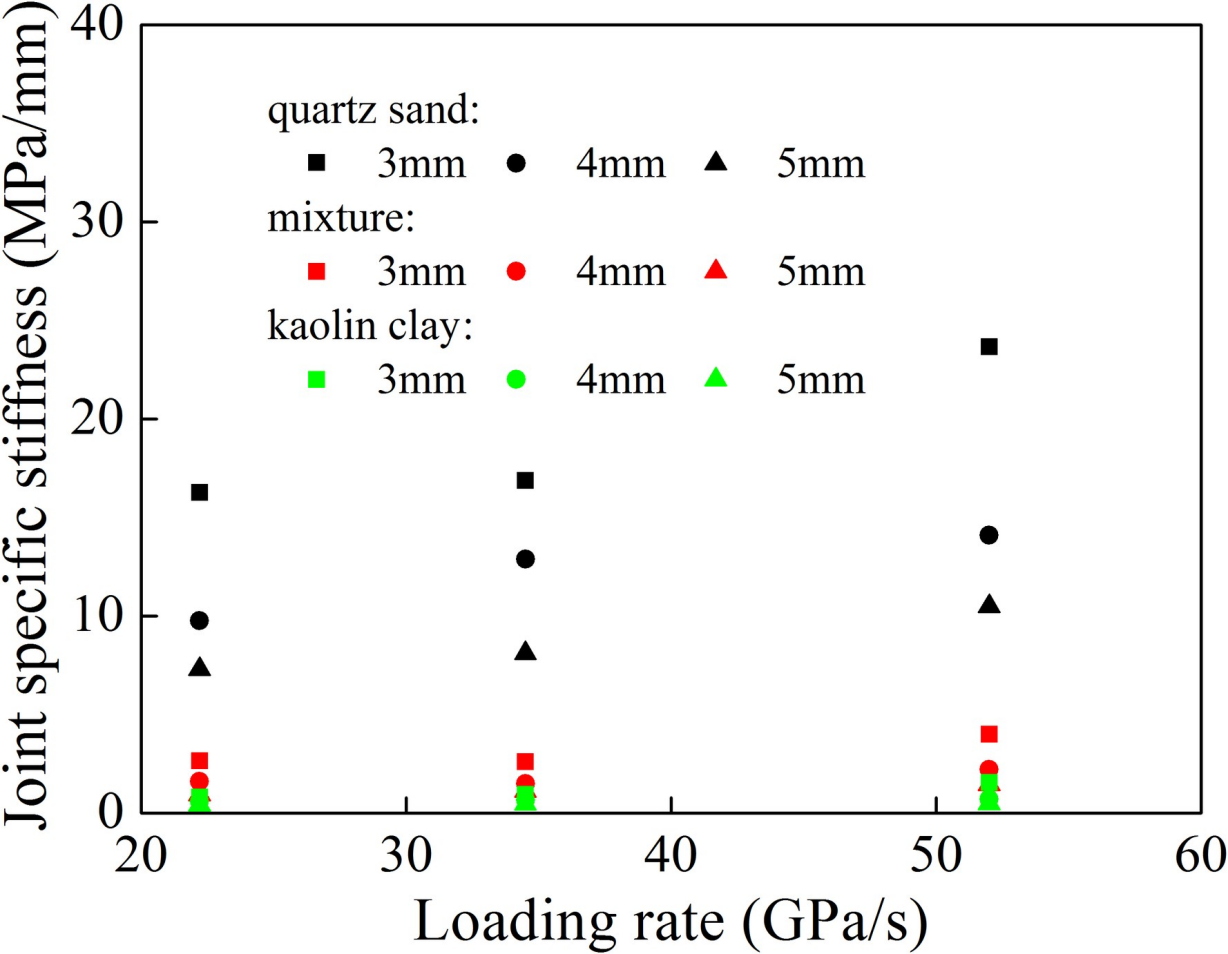
Variation of the joint specific stiffness with the loading rate.

The pre-peak linear slope of the stress-closure curve is defined as the joint specific stiffness. Herein, the relationship between joint specific stiffness and loading rate was analyzed. Fig 14 shows the relationship between joint specific stiffness and loading rate with regard to different filling materials. This figure shows that the joint specific stiffness increased with the increase of the loading rate when the joint was filled with the same materials, and smaller joint thickness led to larger growth of joint specific stiffness. At the same loading rate, the joint specific stiffness was the largest for quartz sand, and the smallest for kaolin clay.

#### 4.2.3 Transmission coefficient

**Fig 15:**
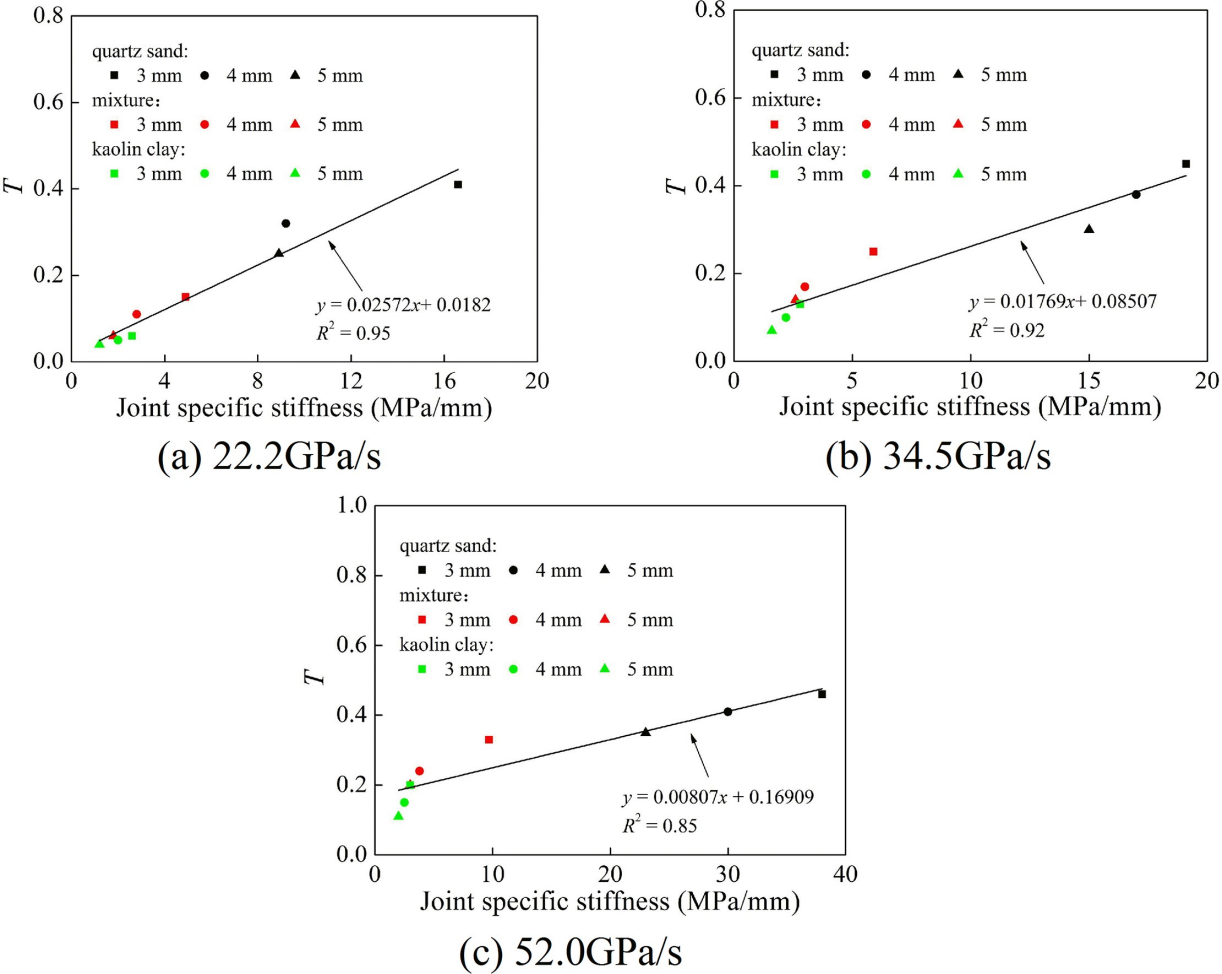
Variation of the transmission coefficient with the joint specific stiffness. (a) 22.2 GPa/s (b) 34.5 GPa/s (c) 52.0 GPa/s.

The filled joint will be deformed under the stress wave. The joint deformation is a kind of energy absorption of stress wave. Therefore, the deformation of the filled joint affects the propagation of stress wave in the jointed rock mass. The wave transmission coefficient is directly measured from the test. In order to study the stress wave propagation characteristics in jointed rock mass, a transmission coefficient is defined as

$$T=\frac{\max[\varepsilon^{p+}(x_{i},t)]}{\max[\varepsilon^{p-}(x_{i},t)]}$$ (21)

where *ε*^p+^(*x*_*i*_,*t*) and *ε*^p−^(*x*_*i*_,*t*) are the positive wave at the joint front and rear end, respectively. Fig 15 shows the relationship between transmission coefficient and joint specific stiffness at different loading rates. Although filling material and joint thickness were different, the transmission coefficient at the same loading rate increased with the increase of joint specific stiffness. They have a linear relationship. Meanwhile, for the same filling material and joint thickness, the transmission coefficient raised with the increase of loading rate.

### 4.3 Test results for two joints

In the rock mass test with two joints, the attenuation degree of stress wave was relatively larger. To ensure that the strain gauges on each rock bar could detect the signal, the loading rate was set as 74.7 GPa/s, 77.8 GPa/s and 84.6 GPa/s, respectively. Meanwhile, the joint set at each loading rate were divided into three joint sets. In the first joint set, both the first joint (*d*_1_) and the second joint (*d*_2_) had 3 mm in width. In the second joint set, *d*_1_ was 3 mm and *d*_2_ was 5 mm. In the third joint set, *d*_1_ was 5 mm and *d*_2_ was 3 mm.

#### 4.3.1 Stress-closure relationship

**Fig 16:**
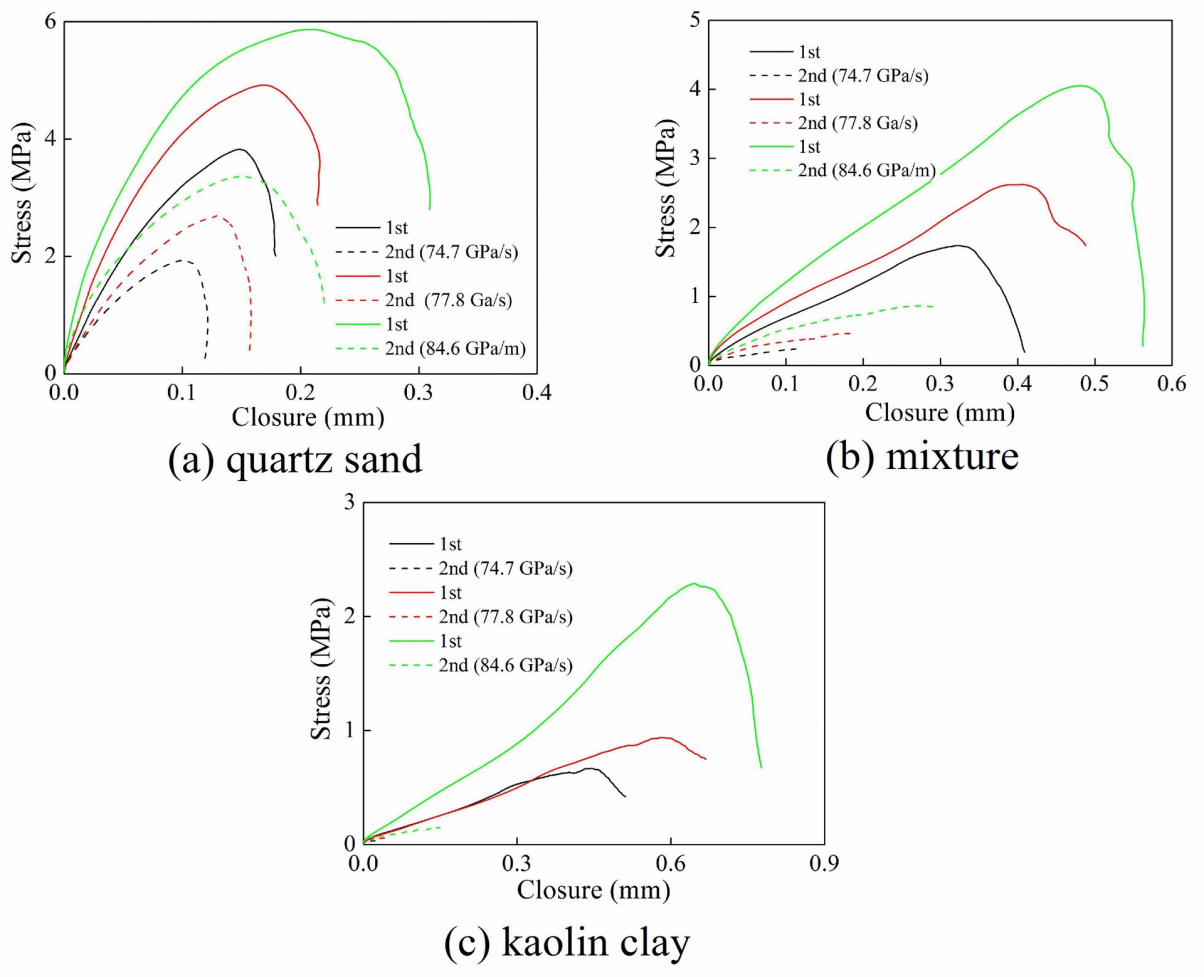
Stress-closure relationship of filled joints with various loading rates. (a) quartz sand (b) mixture (c) kaolin clay.

Fig 16 shows the relationship between stress and joint closure at different loading rates. The width of the joints was 3 mm. It is noticed that the slope of the stress-closure curve gradually decreased in the middle and late loading stage when the filling materials were different. The filling material particles gradually slide or break, and the load transfer capacity gradually decreased. At the same time, the stress-closure curve of the first joint showed “softening” characteristics, while the stress-closure curve of the second joint was different. The stress-closure curve of the second joint showed "softening" characteristic when the joint was filled with quartz sand. For the filling material of mixture and kaolin clay, the stress wave attenuation degree was larger when the stress wave passed through the first joint, resulting in a less evident "softening" characteristic of the second stress-closure curve.

#### 4.3.2 Joint specific stiffness

**Fig 17:**
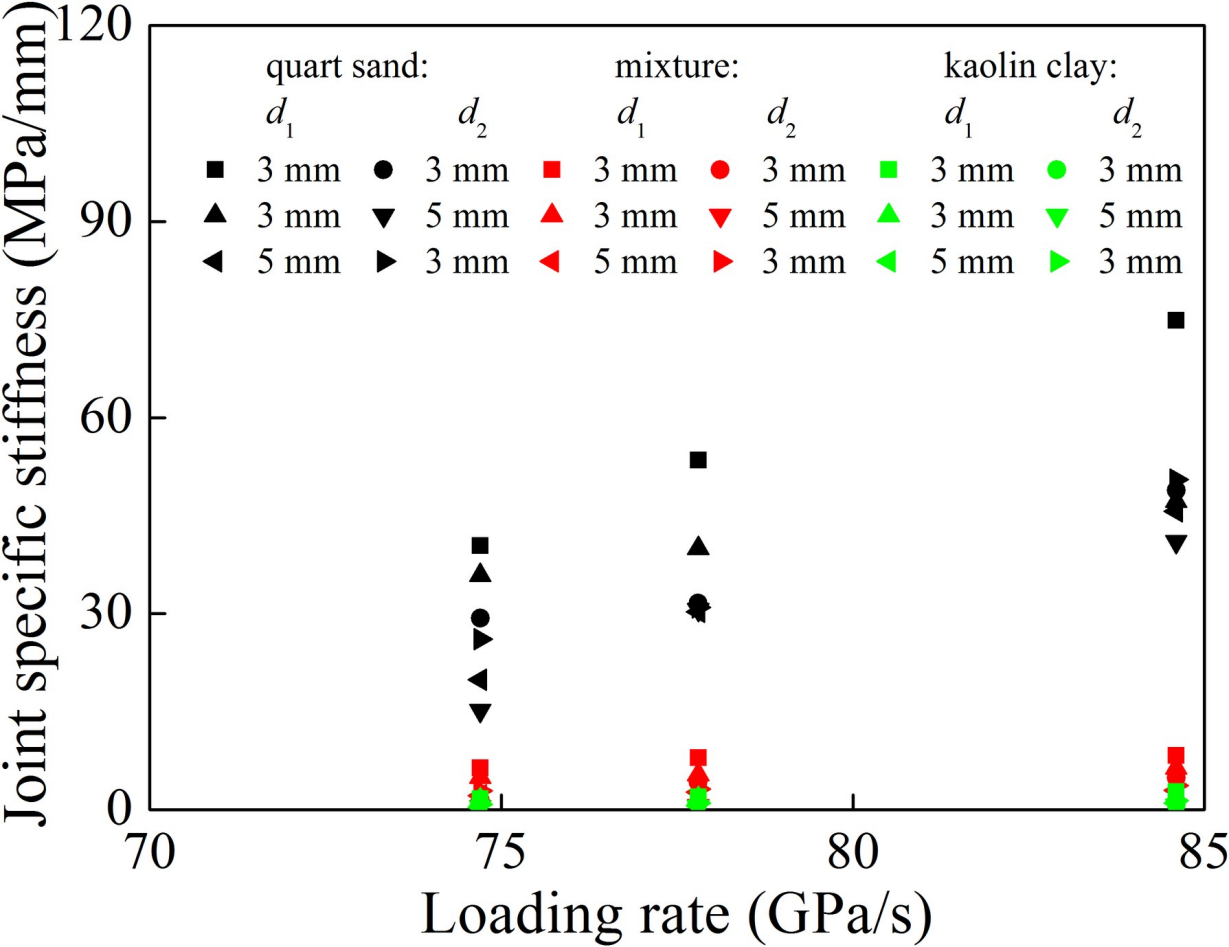
Variation of joint specific stiffness with the loading rate.

Fig 17 shows the relationship between joint specific stiffness and loading rate in different joint set. The joint specific stiffness of each joint set increased with the increase of loading rate. For the filled quartz sand, the joint specific stiffness under each joint set was the largest. When the filling material became mixture or kaolin clay, the joint specific stiffness of mixture in each joint set was greater than that of kaolin clay. Under the same filling material, for the first joint set (*d*_1_ = *d*_2_), the specific stiffness of the second joint was smaller than that of the first one. For the second joint set (*d*_1_ < *d*_2_), the specific stiffness of the second joint was significantly lower than that of the first joint. In the third joint set (*d*_1_ > *d*_2_), the specific stiffness of the second joint was greater than that of the first joint. This is mainly related to the change of loading rate and dominant frequency. For the first and second joint set, when the stress wave passed through the first joint, the loading rate and dominant frequency decreased. This led to the reduction of the joint specific stiffness of the second joint. In the third joint set, the attenuation amplitude of loading rate and dominant frequency of stress wave passing through the first joint was larger than that of the second joint. It resulted in an increase in the specific stiffness of the second joint. Therefore, the joint specific stiffness in each joint set was related to the loading rate and filled joint width. For the same filling material, when the joint width was the same, the joint specific stiffness of the two joints in the joint set was different due to the change of the dominant frequency of the stress wave.

#### 4.3.3 Transmission coefficient

**Fig 18:**
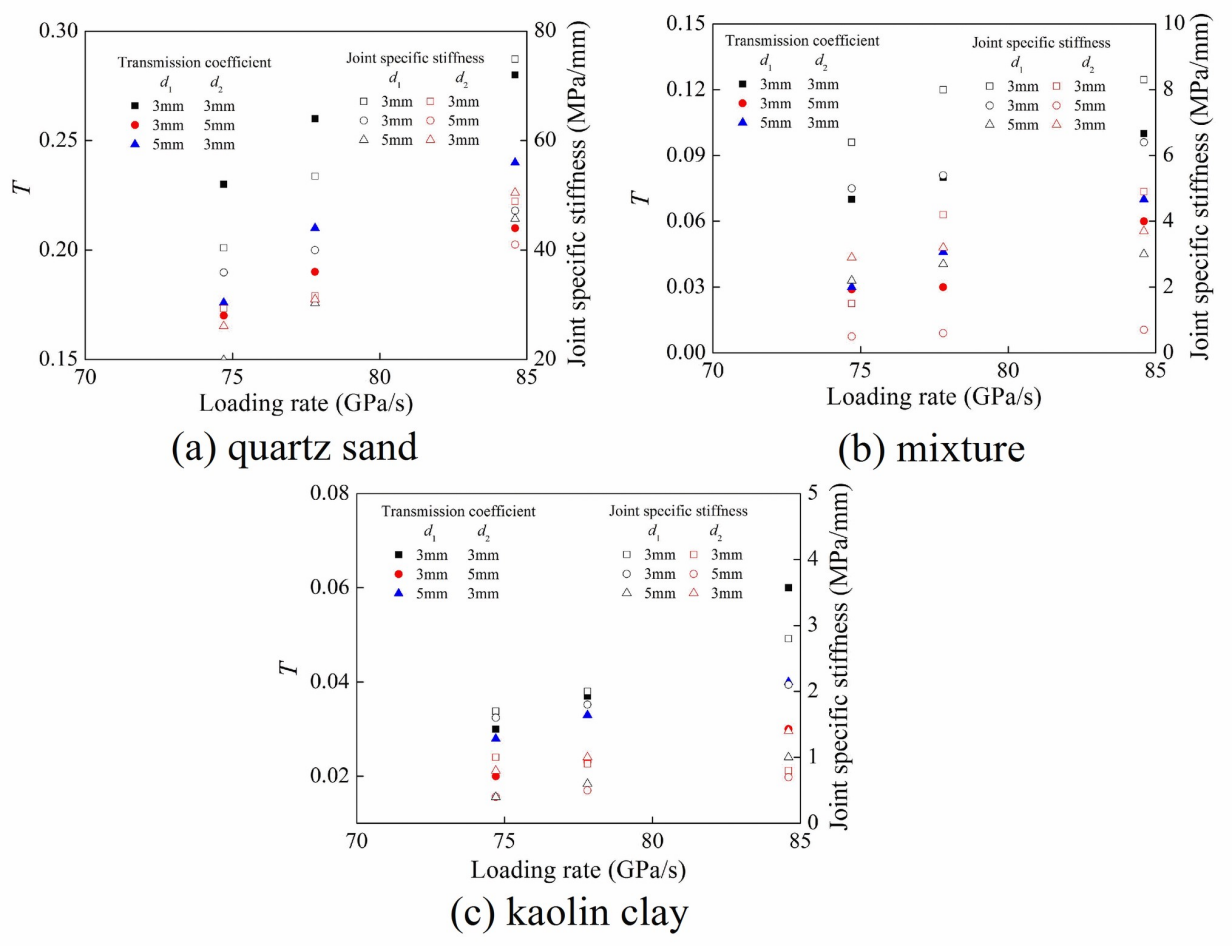
Variation of transmission coefficient with the loading rate. (a) quartz sand (b) mixture (c) kaolin clay.

Fig 18 shows the variation rule of transmission coefficient under different loading rates. The transmission coefficient of each joint set increased with the increase of loading rate. At the same loading rate, the quartz sand filling material dissipated less energy of stress wave, and the transmission coefficient was larger under each joint set. The crushing of kaolin clay particles consumed more energy, leading to the greater attenuation of stress wave, thus the transmission coefficient was reduced.

## 5. Comparison between the analytic and test results

### 5.1 Rock mass with a single joint

**Fig 19:**
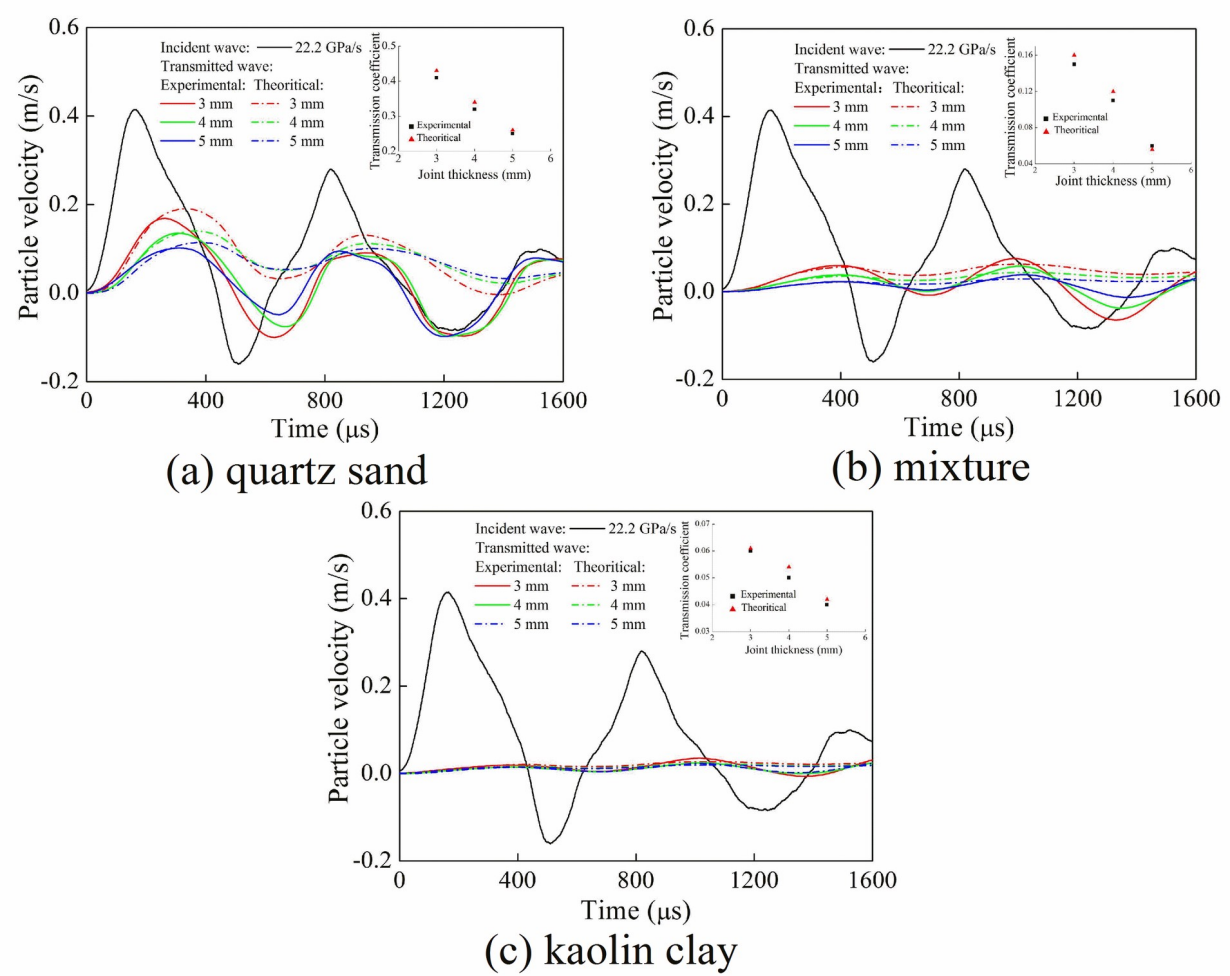
Time-history curves of particle velocity of transmitted waves under different joint widths. (a) quartz sand (b) mixture (c) kaolin clay.

When the stress wave propagates in the jointed rock mass, the ability of the stress wave to pass through the joint is generally concerned. In practical situation, the transmission coefficient is used to describe the ability of stress wave to pass through the joint. Substituting the joint specific stiffness of a single filled joint into Eqs (10) and (11), the particle velocity of transmitted wave through a single filled joint can be calculated. Fig 19 shows the time-history curve of the particle velocity of transmitted wave under different joint widths. It is seen that when the loading rate was fixed, compared with the experimental and the theoretical calculation results, the peak values of the particle velocity of transmitted wave were basically similar, and the transmission coefficient was close. This indicated that the discontinuous displacement model was applicable for filled joint, and the joint specific stiffness could be used to denote the equivalent normal stiffness. For the filling material of mixture and kaolin, due to the fragmentation of filling material particles, the load transfer capacity of particles and the peak value of the particle velocity of transmitted wave were significantly reduced. Meanwhile, the waveforms of transmitted wave obtained by the experiment and theoretical calculation were significantly different. The reason is that the stress wave was reflected at the end of the transmitted bar and then superimposed with transmitted wave in the transmitted bar. However, the reflection of stress wave through the filled joint was not considered in the theoretical calculation.

### 5.2 Rock mass with two joints

**Fig 20:**
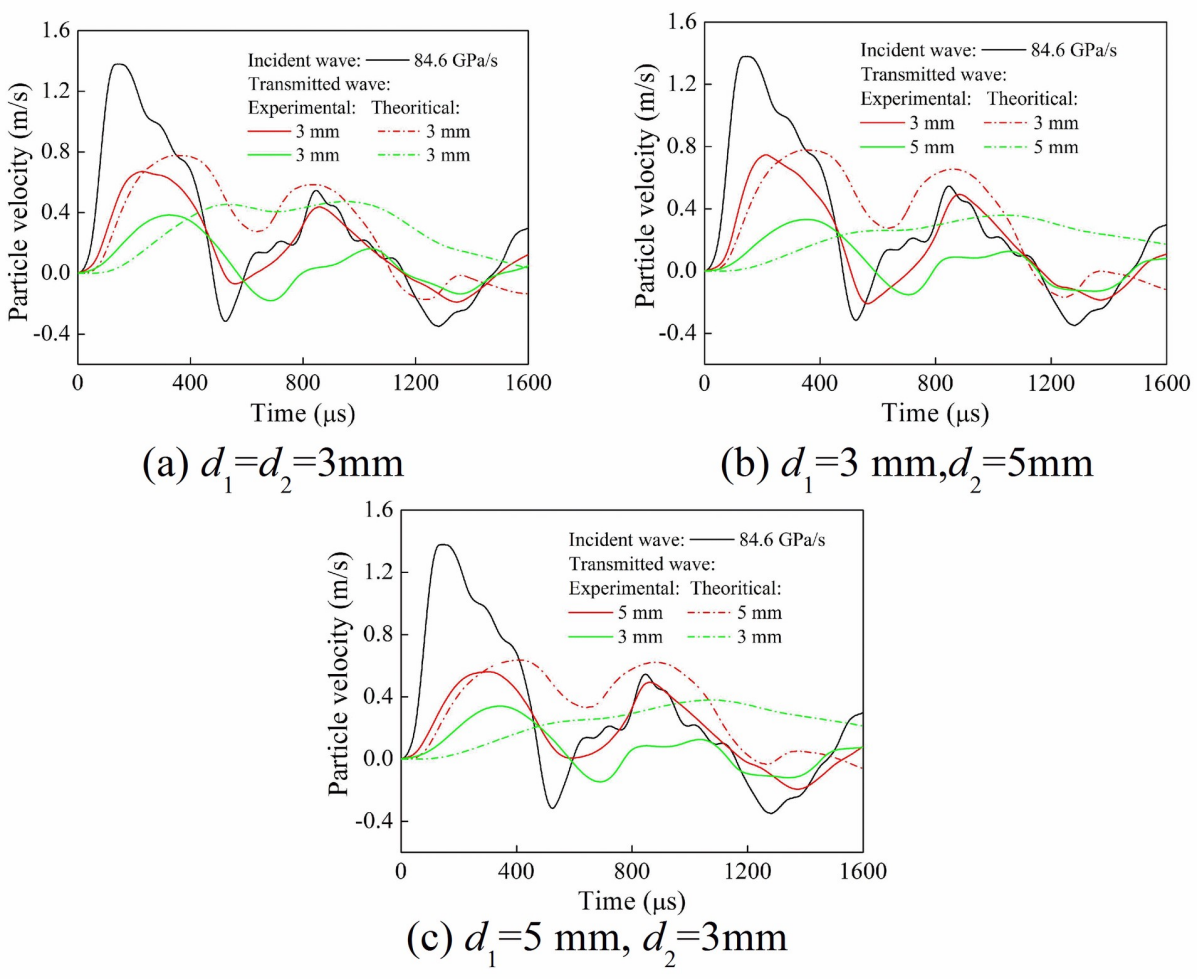
Time-history curves of particle velocity of transmitted waves (quartz sand). (a) *d*_1_ = *d*_2_ = 3 mm (b) *d*_1_ = 3 mm, *d*_2_ = 5 mm (c) *d*_1_ = 5 mm, *d*_2_ = 3 mm.

Fig 20 shows the time-history curves of the particle velocity of transmitted wave. Compared with the experimental and theoretical calculation results, it can be seen that the peak values of the particle velocity of the first and second transmitted waves were relatively close. It indicates that the transmission coefficient had little difference. Meanwhile, the waveform of the first transmitted wave was similar, while the waveform of the second transmitted wave was significantly different. This is mainly related to the superposition of the reflected wave and the transmitted wave. In the theoretical calculations and the experiments, the reflection of stress wave was considered in the first transmitted wave. However, the reflection of stress wave in the theoretical calculations was not considered in the second transmitted wave. In the calculation process of stress wave passing through multiple filled joints, each filled joint could be equivalent to a discontinuous structural surface without considering the joint thickness, and the equivalent normal stiffness could be obtained by the stress-closure curve. During the propagation of stress wave in the filled joint set, the filling material, joint width and loading rate affected the equivalent normal stiffness, and the magnitude of equivalent normal stiffness for each joint was different.

## 6. Discussion

Based on the time-domain recursion method, the propagation equation of stress wave in the filled joint set was deduced, and the filled joint was considered as the structural plane without thickness. It is seen that the theoretical results are in good agreement with the experimental ones, and the propagation characteristics of stress wave in filled joint set can be characterized by the joint specific stiffness. Multiple reflections of stress wave between filled joints affect the propagation of stress wave in the filled joint set. In order to accurately predict the propagation law of stress wave, the specific stiffness of each joint in the filled joint set needs to be considered.

Rock joints are usually filled with sand, clay and other geomaterials. An insight understanding and description of the dynamic properties of filled rock joints is essential for the analysis and design of rock engineering problems under dynamic loads. When the filling material is easily broken, it will consume the energy of stress wave during the particles crushing process. Meanwhile, the load-transfer capacity between the particles drops, resulting in a significant attenuation of stress wave. If the filling materials is not easily destructible, the attenuation degree of stress wave is thus small.

The filled joints with the similar specific stiffness probably have different thicknesses. As for the filled joints with the same thickness, the attenuation of loading rate and dominant frequency may change after the stress wave propagate across the first joint, causing a decrease in specific stiffness of the second joint (refer to Fig 17). Therefore, under the same thickness of filled joints, the joint specific stiffness of the filled joint set decreases with the increase of the filled joints number. In the past studies, each joint was assumed to have the similar mechanical properties and thickness in the joint set, and the effect of wave attenuation on the joint specific stiffness was not given enough considerations [9–11, 30]. However, the transmission coefficient is related to the specific stiffness of each joint in the joint set. This assumption may cause an overestimation of the transmission coefficient.

## 7. Conclusions

The propagation equation of stress wave in the filled joint set was deduced by the time-domain recursion method. Meanwhile, the relationship among stress-closure curve, joint specific stiffness, transmission coefficient and loading rate were analyzed by SHRB tests. The following conclusions are drawn:

1. When the stress wave propagates in the filled joint set, the filled joint is equivalent to a continuous stress and displacement model. The theoretical analysis results are in good agreement with the experiment ones, indicating that the model can well describe the propagation of stress wave in the filled joint set, and the equivalent normal stiffness in the model denotes the joint specific stiffness.
2. For the test of a single jointed rock mass, the joint specific stiffness increases with the increase of loading rate. At a fixed loading rate, the joint specific stiffness is the largest for quartz sand, while the smallest for kaolin clay. In dual joint tests, both the loading rate and dominant frequency change because of the multiple reflection and transmission of stress wave, leading to the variation of joint specific stiffness.
3. For the rock mass with a single joint, the transmission coefficient is linearly related with joint specific stiffness regardless of filling materials. In the rock mass with two joints, the specific stiffness of each joint affects the transmission coefficient, and the transmission coefficient of each joint set increases with the higher loading rate.

## Supporting information

S1 File (M)
